# Comparative Evaluation of Push-Out Bond Strength of Conventional Mineral Trioxide Aggregate, Biodentine, a Modified Mineral Trioxide Aggregate, and Two Novel Antibacterial-Enhanced Mineral Trioxide Aggregates

**DOI:** 10.7759/cureus.56320

**Published:** 2024-03-17

**Authors:** Arokia Rajkumar Shancy Merlin, Vignesh Ravindran, Ganesh Jeevanandan, Rajalakshmanan Eswaramoorthy, Abirami Arthanari

**Affiliations:** 1 Department of Pediatric and Preventive Dentistry, Saveetha Dental College and Hospitals, Saveetha Institute of Medical and Technical Sciences, Saveetha University, Chennai, IND; 2 Center of Molecular Medicine and Diagnostics, Department of Biochemistry, Saveetha Dental College and Hospitals, Saveetha Institute of Medical and Technical Sciences, Saveetha University, Chennai, IND; 3 Department of Forensic Odontology, Saveetha Dental College and Hospitals, Saveetha Institute of Medical and Technical Sciences, Saveetha University, Chennai, IND

**Keywords:** mineral trioxide aggregate, biodentine, pushout bond strength, doxycycline, metronidazole

## Abstract

Background

The challenges associated with incorporating antimicrobial agents, such as the potential diminishment of the cement's physical properties, highlight the need for comprehensive evaluations. Balancing antimicrobial efficacy with the maintenance of structural integrity is a crucial aspect of material development. The acknowledgment of cytotoxic properties associated with tricalcium aluminate, a major constituent in conventional mineral trioxide aggregate (MTA), is critical in terms of long-term evaluation of treatment procedures. The primary focus of the push-out test is to evaluate the resistance of the tested material to dislodgement. Greater push-out strength implies stronger adhesion between the tested material and the tooth surface.

Aim

This study aims to evaluate the push-out bond strength of two antibacterial-enhanced MTAs with conventional MTA and Biodentine.

Material and methods

A total of five materials were tested: a) modified MTA, b) doxycycline-enhanced MTA, c) metronidazole-enhanced MTA, d) conventional MTA, and e) Biodentine. All the materials were mixed based on a predetermined powder:liquid ratio and then carried using a plastic instrument to the desired experimental design. Single-rooted permanent teeth, preferably incisors, were used in the present study. Teeth were embedded vertically in a rubber mold, and sectioning of the tooth was performed. A single operator instrumented the canal space in each slice using Gates-Glidden burs, and the mixed cements were placed in the respective groups and stored for 72 hours. A push-out test was carried out using a universal testing machine. Following the bond failure, the slices were examined under a stereomicroscope to determine the nature of the bond failure. The collected data was subjected to a one-way analysis of variance test, post hoc test, and chi-square test for statistical analysis.

Results

The mean push-out bond strength was found to be the highest for Biodentine (43.25 ± 0.62 megapascals (MPa)), followed by doxycycline- and metronidazole-enhanced MTAs (39.54 ± 0.65 MPa and 39.29 ± 0.16 MPa, respectively), modified MTA formulation (37.75 ± 0.73 MPa), and the lowest for conventional MTA (25.93 ± 0.7 MPa). Conventional MTA samples had an adhesive failure (89.4%), while Biodentine samples had a cohesive failure (80.3%). Mixed failures were noticed with the samples containing modified MTA formulation (71.3%), doxycycline-enhanced MTA (76.6%), and metronidazole-enhanced MTA (78.0%).

Conclusion

Despite not surpassing Biodentine in bond strength, antibacterial-enhanced MTAs are considered potential alternatives to conventional MTA in day-to-day clinical practice.

## Introduction

The push-out bond strength test conventionally assesses the material's resistance to displacement by applying a perpendicular force to the material interface. This test is employed to gauge the interfacial shear strength between various cement-like materials (such as root-end filling, perforation repair material, obturation material, and root canal sealer) and the adjacent tooth structure [1]. This test provides information about the adhesive properties of the material being tested by helping the dental practitioner understand how well the material can bind to the tooth structure. The primary focus of the push-out test is to evaluate the resistance of the tested material to dislodgement. Greater push-out strength implies stronger adhesion between the tested material and the tooth surface. High push-out strength is desirable in clinical applications as it suggests better stability and longevity of dental restoration [2].

Efforts have been made to reduce the cytotoxicity of mineral trioxide aggregate (MTA), improve handling properties, establish better sealing ability, and enhance biocompatibility [3]. The ability of MTA to induce the formation of dentin makes it a valuable material for various pulp therapies, including pulp capping and apexification procedures. The acknowledgment of cytotoxic properties associated with tricalcium aluminate, a major constituent in conventional MTA, is critical in terms of long-term evaluation of treatment procedures [3,4]. Identifying and addressing such issues are crucial for maintaining the biocompatible nature of the cement, which is essential for dental practitioners.

Efforts to enhance the antimicrobial effectiveness of MTA through the incorporation of agents, such as chlorhexidine gluconate, tetracycline, nitric oxide-releasing compounds, calcium hydroxide, fluorohydroxyapatite, and calcium fluoride, are significant [5-7]. However, it is important to strike a balance between enhanced antimicrobial properties and maintaining the desired physical characteristics of the cement. The challenges associated with incorporating antimicrobial agents, such as the potential diminishment of the cement's physical properties, highlight the need for comprehensive evaluations. Balancing antimicrobial efficacy with the maintenance of structural integrity is a crucial aspect of material development.

The investigation into antimicrobial agents, like metronidazole and doxycycline, presents an interesting avenue for research. Metronidazole's potential to effectively combat gram-positive anaerobes, like *Enterococcus faecalis*, adds significance to its consideration in dental applications. However, the limited research available on doxycycline and its impact on the antimicrobial properties of final-set Biodentine cement underscores the necessity for further exploration in this field [8]. The focus on evaluating marginal adaptations and push-out bond strength of bioactive bioceramic cements, especially those augmented with antimicrobial agents, is vital for comprehending their efficacy in clinical scenarios. Therefore, this study aimed to assess the push-out bond strength of three modified MTAs compared to conventional MTA and Biodentine. The null hypothesis tested whether there was no disparity in the push-out bond strength among the various calcium silicate-based materials examined. The objectives of the study were to assess the push-out bond strength of a modified MTA and two antibacterial-enhanced MTAs compared to conventional MTA and Biodentine and to perform a scanning electron microscopy (SEM) analysis to evaluate the failure patterns associated with these materials during the push-out bond strength test.

## Materials and methods

This in-vitro study was designed and performed in the dental material research laboratory of a dental institute. The experimental protocol, design, and composition of the materials used were assessed and approved by the members of the institutional ethical committee.

Material sample preparation

A modified MTA, two antibacterial-enhanced MTAs, and two commercially available bioceramics were used in the present study.

Group 1: Modified MTA Formulation

The composition includes the common elements of silicate cements like tricalcium silicate, dicalcium silicate, calcium carbonate, calcium sulfate, and calcium fluoride as the fundamental powder components. Tricalcium silicate and dicalcium silicate were synthesized in the laboratory following the manufacturing procedure proposed in a previously published article [4]. Calcium chloride powder was sourced from Tokyo Chemical Industry (TCI) Chemicals (India) Pvt. Ltd. (Chennai, India). In the formulation of the liquid component, calcium chloride was blended with 1 mL of distilled water to attain a 20% concentration. The specifics of the powder composition are outlined in Table 1.

**Table 1:** Composition of powder contents of the modified mineral trioxide aggregate and the two antibacterial-enhanced mineral trioxide aggregates used in the current study

Group 1: Modified MTA formulation		Group 2: Doxycycline-enhanced MTA		Group 3: Metronidazole-enhanced MTA	
Powder	Weight percentage (wt%) for every 100 mg of powder	Powder	Weight percentage (wt%) for every 100 mg of powder	Powder	Weight percentage (wt%) for every 100 mg of powder
Tricalcium silicate	55	Tricalcium silicate	60	Tricalcium silicate	60
Dicalcium silicate	30	Dicalcium silicate	20	Dicalcium silicate	20
Calcium fluoride	5	Calcium fluoride	5	Calcium fluoride	5
Calcium sulfate	5	Calcium sulfate	5	Calcium sulfate	5
Calcium carbonate	5	Calcium carbonate	4	Calcium carbonate	4
Zirconium oxide	1	Doxycycline	5	Metronidazole	5
-	-	Zirconium oxide	1	Zirconium oxide	1

A blend consisting of 100 mg of the designated powder content and 40 µl of the liquid component was placed onto a pad. After complete hydration, the mixing process proceeded until a uniform consistency suitable for molding was reached.

Group 2: Doxycycline-Enhanced MTA

The base powder composition for this group was the modified MTA formulation used in Group 1. Doxycycline hyclate and calcium chloride powder were procured from TCI Chemicals (India) Pvt. Ltd. Doxycycline was incorporated in powder form. The liquid component was created by combining calcium chloride with 1 mL of distilled water to achieve a 20% concentration. The specifics of the powder composition are outlined in Table 1. A blend of 100 mg of the specified powder content and 40 µl of the liquid component was deposited onto a pad. Following complete hydration, the mixture was thoroughly homogenized until a uniform consistency suitable for molding was attained.

Group 3: Metronidazole-Enhanced MTA

Similar to Group 2, the modified MTA formulation from Group 1 was used as the base powder composition. Metronidazole and calcium chloride powder were obtained from TCI Chemicals (India) Pvt. Ltd. Metronidazole was incorporated in powder form. The liquid component was created by blending calcium chloride with 1 mL of distilled water to achieve a 20% concentration. The specifics of the powder composition are outlined in Table 1. A blend of 100 mg of the specified powder content and 40 µl of the liquid component was deposited onto a pad. Following complete hydration, the mixture was thoroughly homogenized until a uniform consistency suitable for molding was attained.

Group 4: MTA Angelus^TM^

MTA was acquired from Angelus (Londrina, PR, Brazil) in a powder-liquid presentation. Adhering to the manufacturer's guidelines, a 3:1 powder-liquid ratio was distributed onto a pad. The powder underwent complete hydration by the liquid until achieving a dense, putty-like texture.

Group 5: Biodentine^TM^

Biodentine was acquired from Septodont (Saint-Maur-des-Fossés, France) in a powder-liquid format. As per the manufacturer's recommendations, five drops of the liquid component were combined with the powder capsule and blended using a mechanical triturator (Dentsply Maillefer, Ballaigues, Switzerland) for about 30 seconds. Upon completion, the material displayed a dense, putty-like texture.

Teeth sample preparation

Single-rooted permanent teeth, preferably incisors, were used in the present study. The teeth were extracted from patients who had a non-carious tooth structure with severe periodontitis condition that had a poor prognosis. Signed consent was obtained from the study participants after they were informed about the use of the extracted teeth for research purposes. The teeth were immersed in 5.25% sodium hypochlorite to remove any remnant hard and soft tissues before the samples were stored in saline until the study was performed. On the day of performing the study, the teeth were visually examined. The presence of any caries, fractures, and cracks led to the exclusion of the tooth sample. A radiographic examination was also done to exclude any aberrant anatomy, pulp stones, internal resorption, and the presence of more than one root canal.

Teeth were embedded vertically in a rubber mold with the use of a mounting device that ensured orientation along the long axis. Epoxy resin was used for mounting purposes (Vertex Orthoplast, Vertex-Dental, Zeist, Netherlands). Sectioning of the tooth was performed perpendicular to the long axis of the tooth at the middle third. The teeth were sliced into 2.0 ± 0.05 mm thick slices using a low-speed diamond disc under continuous water irrigation. A total of 120 root dentin slices (n = 24 per group) were obtained.

To standardize the teeth samples used in the study, a single operator instrumented the canal space in each slice using Gates-Glidden burs (Dentsply Maillefer, Ballaigues, Switzerland). A complete pass of burs from size 2 to size 6 sequentially was done to achieve a diameter of 1.5 mm. The prepared samples were rinsed using 17% ethylenediaminetetraacetic acid for 3 min to remove any smear layer formed during the abovementioned canal preparation. Finally, the samples were rinsed using saline, and paper points were used to absorb any remnant liquid along the canal walls. The samples were randomly divided into five groups based on the abovementioned materials prepared. All five prepared materials were mixed as per the instructions provided and placed in the lumen of the slices, which were then condensed using an endodontic plugger. Excess material was removed, and the specimens were wrapped in a piece of wet gauze kept at 100% relative humidity in an incubator at 37°C for 72 hours.

Push-out test

A universal testing machine (Instron Model 5965, Illinois Tool Works (ITW) Inc., Norwood, MA) was utilized during the push-out test. Samples were positioned on a metal slab featuring a central hole to allow unrestricted movement of the plunger, which had a diameter of 1.2 mm. The equipment applied a steady vertical pressure at a rate of 1 mm/min. Precise positioning ensured that the plunger tip made contact exclusively with the test material. Testing continued until complete bond failure occurred. The highest force exerted on the materials upon dislodgement was quantified in newtons. The individual conducting the measurements remained unaware of the association between samples and materials. Push-out bond strength was determined in MPa using the following formula: bond strength (MPa) = force for dislodgement (N) / bonded surface area (mm^2^). The bonded surface area was computed as 2 × π × r × h, where π represents the constant 3.14, r denotes the radius of the root canal, and h signifies the thickness of the dentin slice in millimeters.

Evaluation of failure patterns

After bond failure during the push-out test, the slices underwent examination under a stereomicroscope at 40× magnification to assess the nature of the bond failure. The types of bond failure were classified as follows: a) adhesive failure at the dentin-material interface; b) cohesive failure within the material; and c) mixed failure, which involves a combination of the preceding two failures. The operator analyzing the slices was unaware of the material type used for each sample.

Statistical analysis

Statistical analysis was performed using SPSS statistical software (version 21; SPSS Inc, Chicago, IL). Shapiro-Wilk normality test was performed to check normal distribution. One-way analysis of variance (ANOVA) test was used to compare means of push-out bond strength for all the different materials tested. Post hoc test was performed for intergroup comparisons. Chi-square test was used to compare any associations between the type of material and type of failure. A p-value of less than 0.05 was considered statistically significant.

## Results

The mean push-out bond strength was found to be the highest for Biodentine (43.25 ± 0.62 MPa) and the lowest for conventional MTA (25.93 ± 0.7 MPa). The mean push-out bond strength of the modified MTA formulation (37.75 ± 0.73 MPa) was better than conventional MTA. However, the antibacterial-enhanced MTAs, either the addition of doxycycline or metronidazole, had higher mean push-out bond strength (39.54 ± 0.65 MPa or 39.29 ± 0.16 MPa, respectively) than the conventional MTA and the modified MTA formulation but still lease than Biodentine. ANOVA showed a statistical significance rejecting the null hypothesis that all the test materials have an equal mean push-out bond strength. Post hoc tests revealed that there were statistically significant differences when comparing the MTA group with all the other groups and the Biodentine group with all the other groups (Table 2).

**Table 2:** Mean push-out bond strength of the test materials used in the present study MTA, mineral trioxide aggregate; MPa, megapascals * Statistically significant when compared with the mean mineral trioxide aggregate group (post hoc test); ✝ Statistically significant when compared with Biodentine group (post hoc test).

Group	Material	Mean ± SD (MPa)	p-value
1	Modified MTA formulation	37.75 ± 0.73*^,^^✝^	0.000
2	Doxycycline-enhanced MTA	39.54 ± 0.65*^,^^✝^	
3	Metronidazole-enhanced MTA	39.29 ± 0.16*^,^^✝^	
4	MTA	25.93 ± 0.7*	
5	Biodentine	43.25 ± 0.62^✝^	

Regardless of the cement used, all types of failures were noticed during the SEM analysis of the samples. Mixed type of bond failures were noticed with the samples containing modified MTA formulation (71.3%), doxycycline-enhanced MTA (76.6%), and metronidazole-enhanced MTA (78.0%). Majority of the samples under conventional MTA had adhesive type of failure (89.4%), while Biodentine samples showed cohesive type of failure (80.3%) (Table 3).

**Table 3:** Types of bond failures of the test materials used in the present study MTA, mineral trioxide aggregate; %, percentage (value of measurement)

Group	Material	Adhesive (%)	Cohesive (%)	Mixed (%)
1	Modified MTA formulation	10.3	18.4	71.3
2	Doxycycline-enhanced MTA	8.2	15.2	76.6
3	Metronidazole-enhanced MTA	7.4	14.6	78.0
4	MTA	89.4	2.5	8.1
5	Biodentine	13.5	80.3	6.2

**Figure 1:**
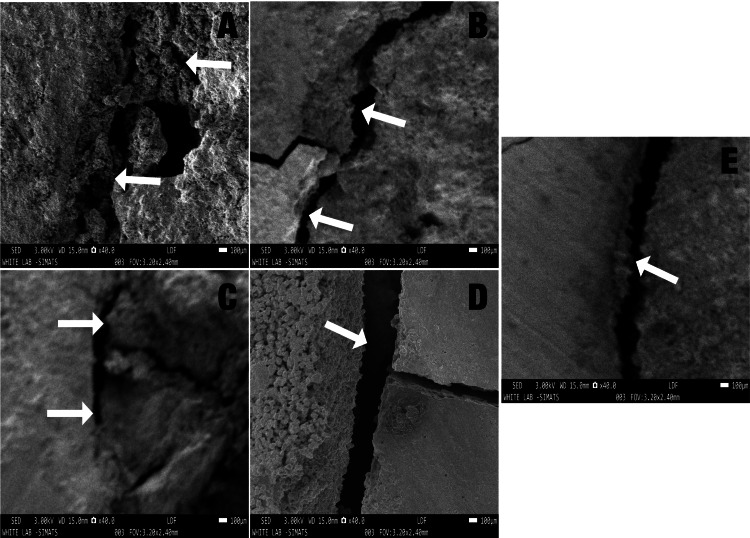
Representative images of the scanning electron microscopic analysis of the predominantly noticed bond failures with the materials tested. A: Mixed failure in group 1 samples; B: Mixed failure in group 2 samples; C: Mixed failure in group 3 samples; D: Adhesive failure in group 4 samples; E: Cohesive failure in group 5 samples, respectively.

Representative images of the SEM analysis are provided in Figure 1. An example of a mixed type of bond failure in a modified MTA sample can be noticed in Figure 1A. An example of a mixed type of bond failure in a doxycycline-enhanced MTA sample can be noticed in Figure 1B. An example of a mixed type of bond failure in a metronidazole-enhanced MTA sample can be noticed in Figure 1C. An example of the adhesive type of bond failure in a conventional MTA sample can be noticed in Figure 1D. An example of the cohesive type of bond failure in the Biodentine sample can be noticed in Figure 1E.

## Discussion

The use of endodontic materials for procedures, such as apical plug in apexification, repair of perforations, and capping during vital pulp therapy, requires these materials to withstand mechanical stresses during tooth function [9]. As mentioned, the evaluation of the adhesion of these materials to dentin surfaces is crucial for assessing their ability to resist mechanical stresses. This adhesion can be measured using different bond strength tests, including tensile, shear, and push-out bond strength.

The consideration of marginal adaptations and push-out bond strength in the assessment of bioactive bioceramic cements is crucial for evaluating their performance in clinical applications. Marginal adaptations and push-out bond strength assessments provide valuable insights into the material's ability to resist dislocating forces and form a reliable bond with dentin [10]. These properties are critical for the success of dental materials in various clinical scenarios. The use of push-out bond strength testing is particularly advantageous because it allows for specimen standardization and replicates clinical stress conditions [11]. The test has been utilized over the past few decades for the assessment of various endodontic materials in clinical practice. This makes the assessment more reliable and practical, providing a closer representation of how the material performs in real-world situations.

There is no published data comparing the push-out bond strength of calcium silicate-based cements with added antimicrobial agents. This highlights a notable gap in the existing literature. This gap underscores the need for research to fill this knowledge void, especially considering the increasing interest in incorporating antimicrobial agents into dental materials. The incorporation of antimicrobial agents to enhance the antimicrobial properties of conventional MTA is a significant consideration in endodontic research. The comparison with Biodentine, a tricalcium silicate cement known for its antimicrobial activity, adds context to the need for improving the antimicrobial characteristics of conventional MTA.

The acknowledgment of low to moderate antimicrobial activity against *E. faecalis* in conventional MTA underscores the importance of addressing this limitation [12]. The addition of agents like calcium fluoride and fluorohydroxyapatite to enhance the antimicrobial activity of MTA is a well-founded strategy [13,14]. The inclusion of 5 wt% of doxycycline and metronidazole in groups 2 and 3, respectively, in the present study reflects a targeted approach to improving the antimicrobial properties of the modified MTA. *E. faecalis*, a highly resistant endodontic pathogen, has been shown to be lethal to nitroimidazole antibiotics, such as metronidazole [15]. Tetracycline group drugs, including minocycline and doxycycline, have a history of use in triple antibiotic pastes [15]. This basis formed the rationale for incorporating metronidazole and doxycycline in the current study.

The use of a universal testing machine for the push-out bond strength test is a standard and widely accepted method in endodontic research. This method allows for the quantification of the material's adhesion to dentin surfaces, providing valuable information about its mechanical stability under different loading conditions [16]. The decision to test the samples at an identical elapsed time of 72 hours is a prudent approach. Standardizing the post-mixing time helps minimize the potential variability in setting times among the different materials used. This ensures a more consistent and reliable assessment of the push-out bond strength.

Comparative studies that evaluate the push-out bond strength of calcium silicate-based cements with and without added antimicrobial agents would be instrumental in understanding the impact of these agents on the material's mechanical properties. This information is essential for clinicians and researchers to make informed decisions about the selection and use of these bioactive bioceramic cements in dental procedures. The results of the present study, indicating that Biodentine had better push-out bond strength compared to conventional MTA and the three modified MTAs, align with findings from previous research [17-19]. This consistency across studies strengthens the validity of the conclusions. It is noteworthy that this superiority was maintained regardless of the presence of a smear layer or other irrigants, emphasizing the robustness of Biodentine's bond strength.

The comparison in a previous study with BioAggregate, an aluminium-free cement, showed a variation in the push-out bond strength [20]. While this study suggested that the absence of tricalcium aluminate in BioAggregate contributed to its lower bond strength, the present study's modified MTAs, also aluminium-free, did not exhibit any compromise in bond strength. This discrepancy might be attributed to the addition of calcium fluoride to the modified MTAs, which potentially influenced their bond strength positively. The positive impact of calcium fluoride on bond strength, as observed in the present study, aligns with findings from another research, where a novel fast-setting calcium silicate demonstrated improved bonding to dentine over time, attributed to the presence of fluoride [21]. This supports the idea that the addition of fluoride can enhance the bond strength of calcium silicate-based cements.

Enhancing the formulation of MTA with the addition of antibacterial agents like doxycycline and chlorhexidine gluconate to enhance the antimicrobial activity of MTA is a well-founded strategy. These agents have been suggested in previous studies and hold promise for improving the effectiveness of the material against microbial challenges but had a compromise in the physical properties [8,22]. Contrasting results in terms of bond strength were exhibited in the present study with the addition of doxycycline or metronidazole to the modified MTA. The results of the present study showed that the enhancement with antibacterial agents slightly improved the push-out bond strength when compared to the modified MTA and significantly improved when compared to conventional MTA. This could be due to the fact that we have added the antibacterial agents in the powder component in the present study while the previous studies attempted to modify the liquid component [8,22]. This statement was supported by the findings of another study that stated that an increase in the powder component in the powder:liquid ratio resulted in an increase in the bond strength [23].

Samples in the conventional MTA group showed adhesive-type failures. Adhesive failures are considered unacceptable for clinical use in dentistry, which implies that the bond between the material and the root dentin was not strong enough. Cohesive-type failures were observed in the Biodentine group suggesting that the material itself maintains integrity, indicating a strong bond within the dentin. The findings of this study were supported by the conclusions of the previous research [24-27]. These studies likely contribute additional evidence and support to the conclusions drawn in the present study regarding the types of bond failures observed with different dental materials. Modified MTA and antibacterial-enhanced MTAs showed a predilection toward mixed-type failures, which are still considered acceptable for clinical use. As this is the first study performed with the addition of antibacterial agents to the powder component, there are no other studies to compare the current evidence.

However, a few limitations have to be addressed. Being an in-vitro experiment, it may still limit the generalizability of findings to real-world oral environments, necessitating validation through clinical trials involving animals and humans. Moreover, the focus on short-term evaluations of push-out bond strength overlooks the crucial aspect of long-term performance of the material in-vivo. Addressing these limitations and conducting additional research could enhance the validity and applicability of the study findings in clinical practice.

The study is noted as the only one found in the literature that specifically assessed push-out bond strength for MTA-based cements with the addition of antibacterial agents. This implies that the study addresses a gap in the existing research by specifically examining the impact of antibacterial agents on push-out bond strength in MTA-based cements. As the present study has an in-vitro methodology, it cannot fully replicate the in-vivo clinical environment. Therefore, the results need to be considered with caution when extrapolating the findings to real-world clinical scenarios, as in-vitro conditions may not fully capture the complexities of the oral environment. Clinical trials involving animals and human subjects are required for a better understanding of the material in the oral environment.

## Conclusions

The results of the present study suggest that, despite not surpassing Biodentine in bond strength, antibacterial-enhanced MTAs are considered potential alternatives to conventional MTA in day-to-day clinical practice. The addition of agents like doxycycline and metronidazole aims to enhance the antimicrobial properties of modified MTAs, addressing limitations against pathogens like *E. faecalis*. Comparative studies would aid in understanding the impact of antimicrobial agents on mechanical properties, essential for informed clinical decisions.
